# Ocular side effects of Trametinib and Dabrafenib: a case report

**DOI:** 10.1186/s12348-023-00339-0

**Published:** 2023-04-12

**Authors:** Bilge Tarım, Meltem Kılıç

**Affiliations:** Department of Ophthalmology, University of Health Sciences, Ankara Bilkent City Hospital, Ankara, Turkey

**Keywords:** Trametinib, Dabrafenib, Uveitis, Cystoid macular edema, Serous retinal detachment

## Abstract

A 53-year-old man who has stage 4 non-small cell lung carcinoma and treated with Dabrafenib-Trametinib combination chemotherapy; presented with decreased bilateral visual acuity. We checked out slit lamp examination, fundoscopy, optical coherence tomography and fundus fluorescein angiography. In slit lamp biomicroscopy; bilateral posterior synechiae, granulomatous keratic precipitates and + 4 cells in the anterior chamber were detected. Cystoid macular edema and subretinal fluid accumulation were revealed in optical coherence tomography. Dabrafenib and Trametinib treatments were discontinued and systemic methylprednisolone, topical corticosteroid and topical cyclopentolate were started. His best corrected visual acuity was increased from counting fingers from 2 m to 0,9 bilaterally and cystoid macular edema and serous retinal detachment were completely regressed as a result of systemic and topical corticosteroid treatment.

## Introduction

One of the most common malignant tumors in human beings is lung cancer and non-small cell lung carcinoma (NSCLC) is the most widespread type of this cancer. Overall, 85% of all lung cancers fall into this category. NSCLC has three main types, determined by the type of cell found in the tumor; adenocarcinoma (ADC), squamous cell carcinoma (SCC) and large cell carcinoma (LCC). Of all lung cancers, 40% are adenocarcinoma, 25–30% squamous cell carcinoma and 10–15% large cell carcinoma [1, 2]. In 1–3.5% of NSCLC, it is found that B-raf proto-oncogene serine-threonine kinase (BRAF) mutations are present. Mutations in the BRAF gene are roughly divided into two, based on their location, at amino acid position 600 and at other positions [3]. Trametinib as MEK inhibitor and Dabrafenib as serine-threonine kinase (BRAF) inhibitor are the chemotherapeutics that can be used in non-small cell lung carcinoma (NSCLC). NSCLCs with V600E mutation are responsive to Dabrafenib while non-V600E NSCLCs are resistant to it [4]. Targeted therapies such as MEK inhibitors are the choices of alternative treatments in non-V600E mutated NSCLCs [5, 6]. It has been shown in the studies that; retinal vein occlusion, serous retinal detachment and central serous retinopathy may occur as a result of Trametinib usage, uveitis and cystoid macular edema may also occur with Dabrafenib use. It has been observed that these ocular side effects are reversible and good response to steroid treatment is obtained [7].

When we look at the literature; although MEK inhibitors and BRAF inhibitors are drugs that are generally used in the treatment of malignant melanoma, the interesting detail in this case is their use in NSCLC [8].

Here, we report a patient who was treated with Dabrafenib-Trametinib combination chemotherapy for stage 4 non-small cell lung cancer and evolved bilateral cystoid macular edema, uveitis and serous retinal detachment as a result of ocular toxicities of the drugs.

## Case report

A 53-year-old man presented with decreased bilateral vision. He was diagnosed with stage 4 non-small cell lung carcinoma; treated with Dabrafenib-Trametinib combination chemotherapy. The patient, who was treated with Dabrafenib-Trametinib combination chemotherapy for 6 months, had a complaint of decreased vision, and the drugs were discontinued for 2 weeks, however there was no improvement in his clinical symptoms. The patient was then referred to our clinic. He had no known systemic disease other than non-small cell lung carcinoma. Cranial and orbital computed tomography results taken in an external medical center were normal.

**Fig. 1:**
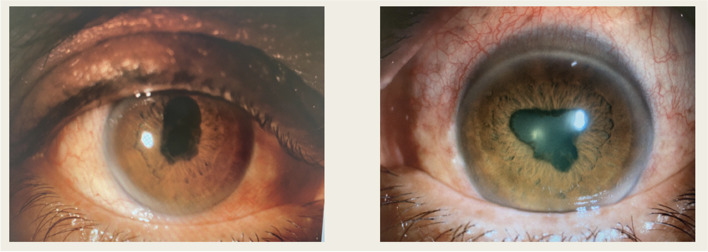
Anterior segment photographs of the right and left eyes at presentation

On our first examination; his best corrected visual acuity was counting fingers from 2 m bilaterally. In anterior segment examination by slit lamp biomicroscopy; there was posterior synechiae, granulomatous keratic precipitates and + 4 cells in anterior chamber, bilaterally (Fig. 1). In dilated fundus examination with cycloplegia, vitritis was present with + 3 vitreous cells and bilateral papilledema was detected. Complete blood count and biochemistry results were normal, except that the white blood cell was 15.66 µl and the neutrophil was 12.75 µl. Also; VDRL, Borrelia IgM, Borrelia IgG and hepatitis markers were negative. Multiple nonspecific gliotic changes in various areas were reported in cranial and orbital magnetic resonance imaging.

Cystoid macular edema and subretinal fluid accumulation were present and retinal thickness was also increased in optical coherence tomography, at presentation. Papilledema and vitritis could be seen in color fundus photographs.

**Fig. 2:**
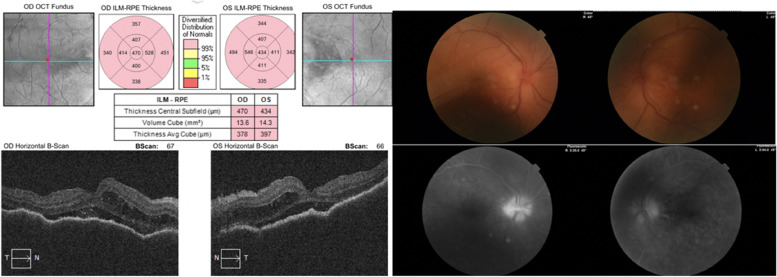
02.09.2021 (At presentation): BCVA (counting fingers from 2 m/counting fingers from 2 m)

**Fig. 3:**
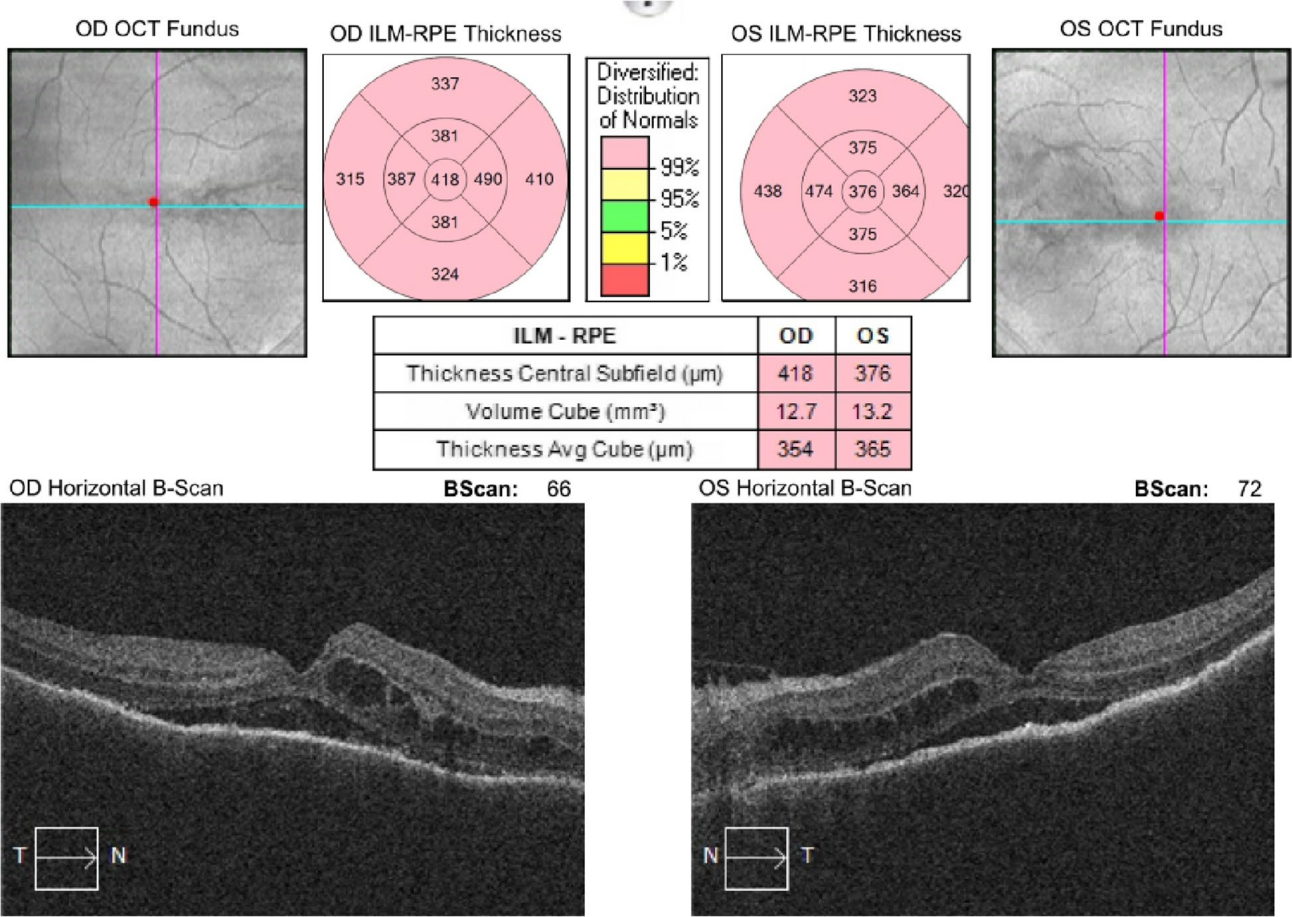
03.09.2021: BCVA (0.1/0.1)

**Fig. 4:**
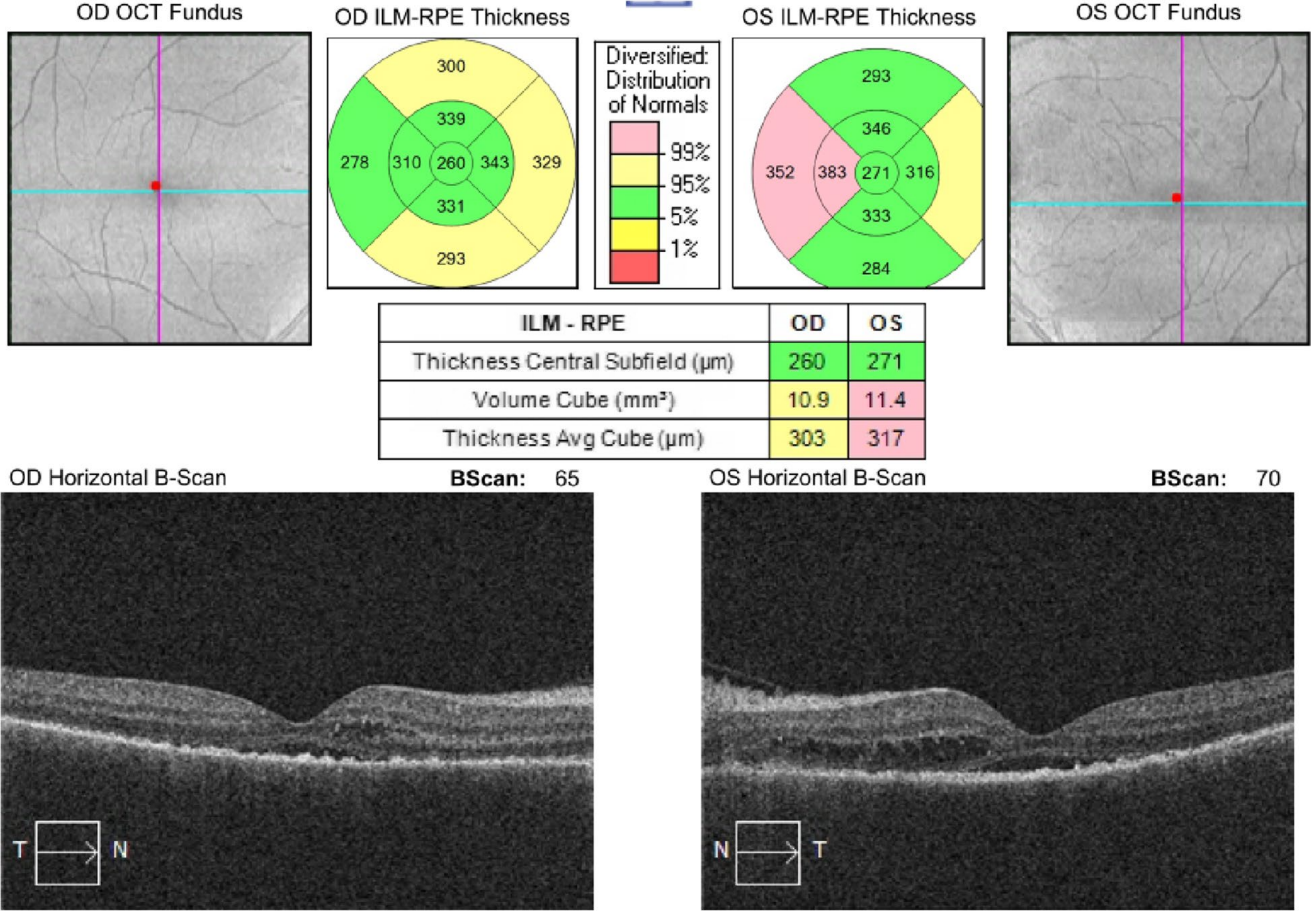
06.09.2021: BCVA (0.4/0.4)

**Fig. 5:**
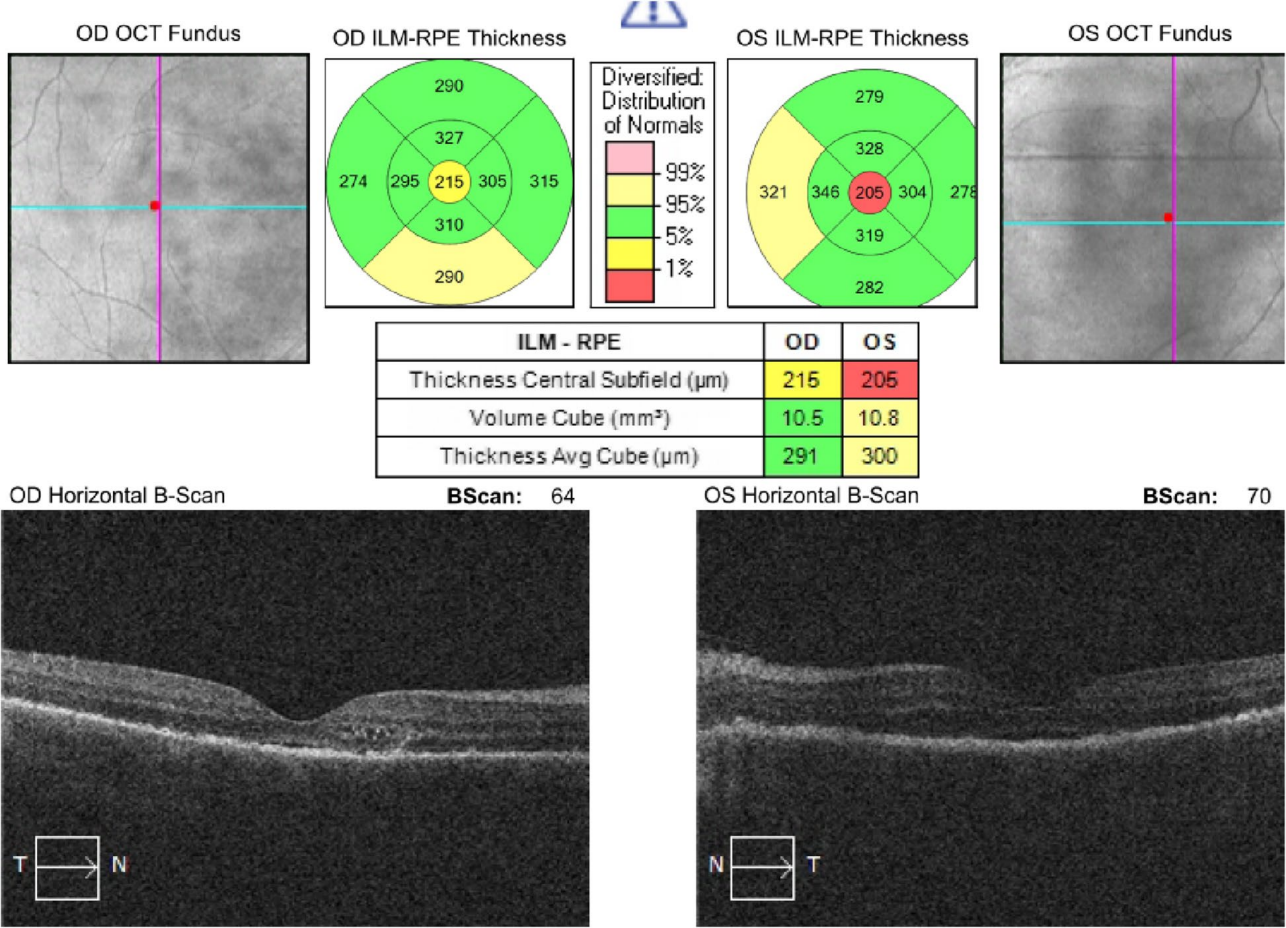
09.09.2021: BCVA (0.4/0.7)

**Fig. 6:**
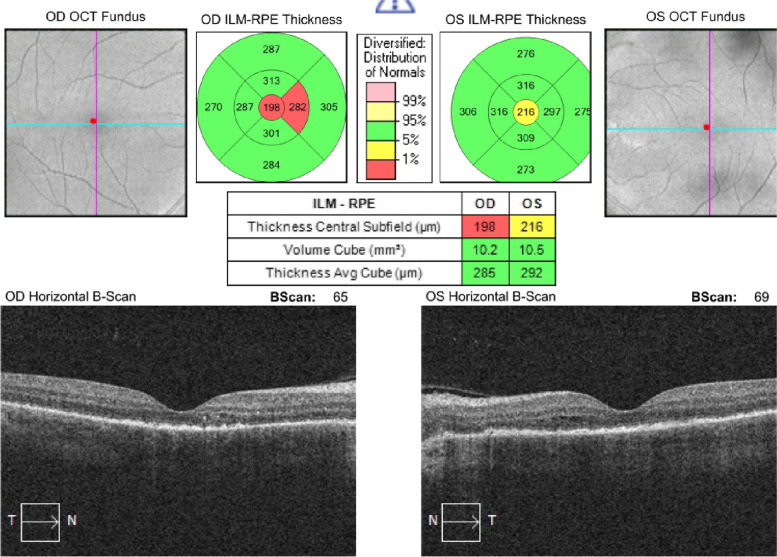
16.09.2021: BCVA (0.8/0.8)

**Fig. 7:**
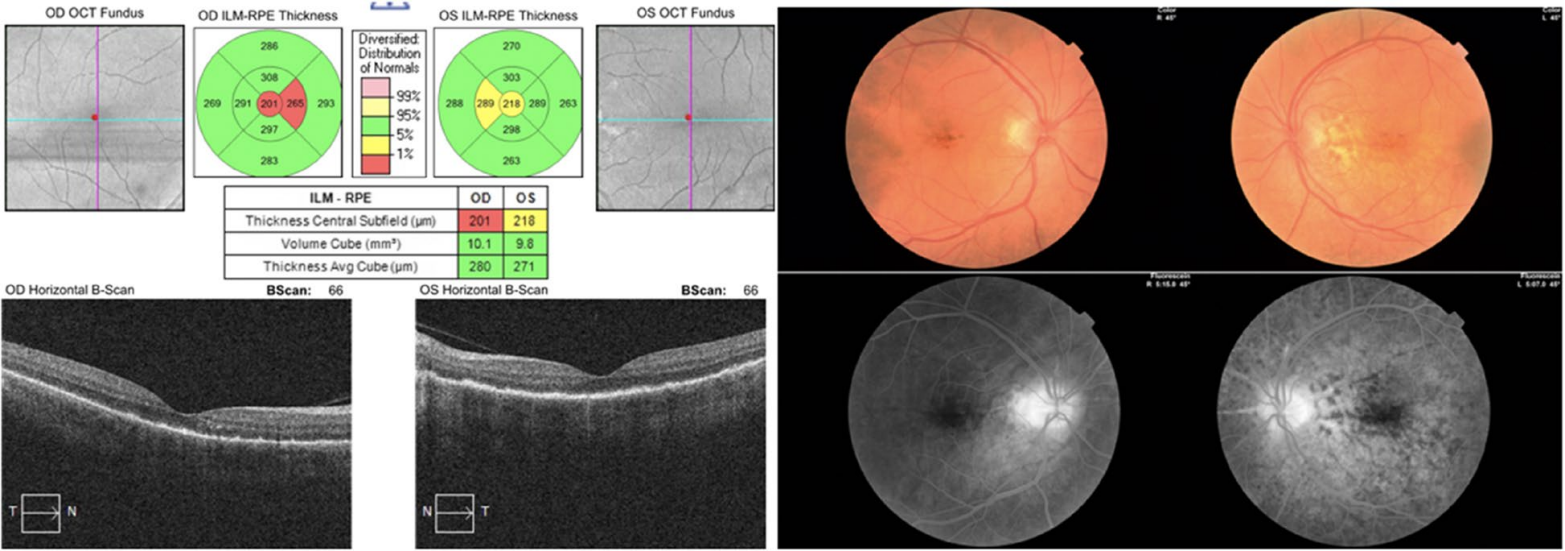
30.09.2021: BCVA (0.9/0.9)

Based on these findings, the ocular side effects of Dabrafenib-Trametinib combination chemotherapy were considered in the patient and 1 gr intravenous methylprednisolone daily for 3 days was started, as well as topical steroid once an hour and cyclopentolate 3 times a day to both of the eyes. After 3 days of intravenous pulse steroid therapy, weekly reduction regimen was applied and 1 mg/kg oral steroid maintenance therapy was started. The result of the systemic and topical steroid we gave to the patient and the cessation of the chemotherapeutics showed that the cystoid macular edema and subretinal fluid in the OCT were completely regressed, and the best corrected visual acuity progressed from counting fingers from 2 m to 0.9 bilaterally with Snellen chart in 28 days. The progression of the patient in optical coherence tomography as a result of the treatment can be seen in the figures below (Figs. 2, 3, 4, 5, 6, 7).

**Fig. 8:**
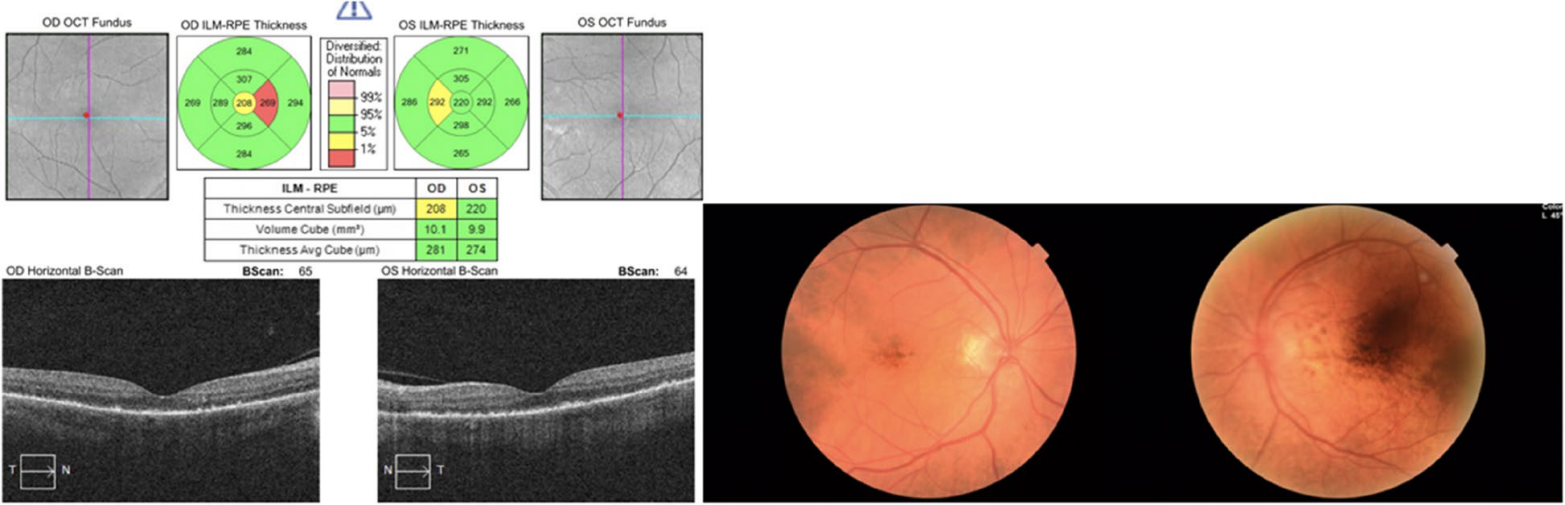
07.10.2021: BCVA (0.4/0.4)

**Fig. 9:**
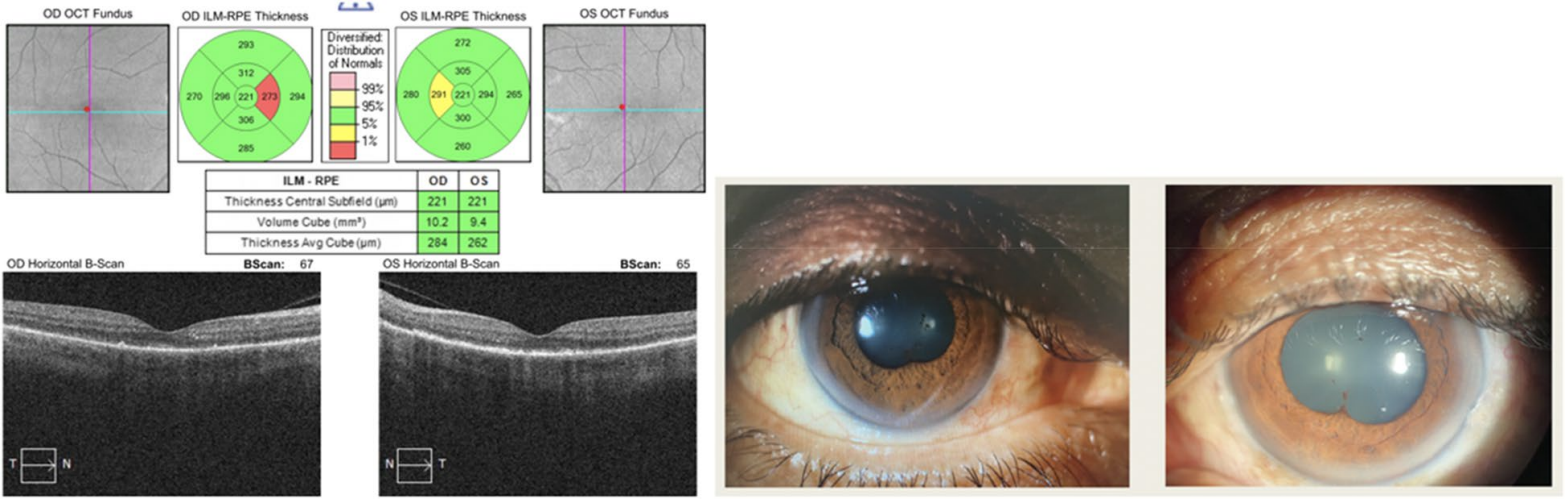
21.10.21: BVCA (0.7/0.7)

When we evaluated the patient 1 week after the last control, we learned that the corticosteroid treatment was stopped and Trametinib treatment was started again, by the patient's oncologist. His best corrected visual acuity was 0.4 bilaterally and there were + 2 cells in anterior chamber and posterior synechiae also bilaterally. In dilated funduscopic examination, there was no clinical evidence of vitritis. The OCT and color fundus photographs are seen in (Fig. 8). The patient was administered on oral and topical corticosteroid treatment again. After 14 days; the patient's visual acuity increased from 0.4 to 0.7, and the + 2 cell in the anterior chamber regressed to + 1 cell bilaterally (Fig. 9).

## Discussion

Trametinib as MEK inhibitor and Dabrafenib as serine-threonine kinase (BRAF) inhibitor are the chemotherapeutics that can be used in non-small cell lung carcinoma (NSCLC). Both Dabrafenib and Trametinib cause inhibition of the MAPK pathway. Molecules responsible for the MAPK pathway are signal transducer kinases that cause cell growth, proliferation and inhibit apoptosis. Under normal conditions, Ras GTPase is activated after dimerization and autophosphorylation after the growth factor binds to the tyrosine kinase receptor on the cell surface. As a result, the RAF oncogene is activated. At the same time, another protein kinase, MEK, is activated and gene expression begins with ERK (Extracellular signal-regulated kinase). Uncontrolled signals in BRAF mutant NSCLC cause tumor growth [3–5]. Recent studies have shown that MEK inhibitors cause subretinal fluid accumulation by causing changes in retinal pigment epithelial permeability. In addition, retinal vein occlusion, macular edema, serous retinal detachment, central serous retinopathy and uveitis were seen to occur as inhibitors of MEK. Among the ocular toxicities of BRAF inhibitors-another chemotherapeutic group-, uveitis and macular edema have been shown [7, 9, 10]. It has been shown that these ocular toxicities are reversible and a good response to corticosteroid treatment is obtained [7].

In a study by Urner-Bloch et al., 32 patients with advanced cutaneous melanoma were treated only with the selective MEK inhibitor binimetinib; or treated in combination with B-RAF inhibitors and it is found that these chemotherapeutics can cause impermanent retinopathy [11]. A case of uveitis as a toxic effect of drugs in a patient with stage 3b cutaneous melanoma treated with Dabrafenib and Trametinib was also published by Joshi et al. [12].

When we reviewed the literature, we found a case report similar to our case [13]. A 66-year-old male patient, who was followed up with the diagnosis of stage 4 cutaneous melanoma, applied with complaints of blurred vision and pain in both eyes. He has been receiving Dabrafenib 75 mg 4 × 1 and Trametinib 2 mg 1 × 1 treatment for 3 months. The best corrected visual acuity was 20/25 in both eyes and bilateral serous retinal detachment was detected in OCT. Worsening of symptoms occurred 2 weeks after discontinuation of given combined chemotherapeutic drugs. His best corrected visual acuity was 20/40 on the right and 20/50 on the left, and + 1,2 cells and posterior synechiae were detected in the anterior chamber; however, vitritis was not observed. Dabrafenib was started again in the patient who showed improvement as a result of topical corticosteroid treatment, and after 1 week, when the symptoms worsened, Dabrafenib was completely discontinued and subtenon steroid injection was administered. After 6 months, Dabrafenib and Trametinib combination chemotherapy was started again and the symptoms and signs recurred [13]. Also, in our case, in the patient followed up with stage 4 NSCLC and treated with Dabrafenib-Trametinib combination chemotherapy, the patient developed bilateral uveitis, cystoid macular edema and central serous retinal detachment. After the chemotherapeutics were stopped and topical and systemic corticosteroid treatment was started, significant improvement was observed in the symptoms and findings. However, upon discontinuation of steroid treatment and re-initiation of trametinib treatment, it was observed that the patient's visual acuity decreased and the findings reappeared.

We can roughly examine uveitis in two classes; including infectious and non-infectious. While it is seen that many different microorganisms can cause infectious uveitis [14]; non-infectious uveitis are autoimmune or immune mediated.

In Vogt-Koyanagi-Harada (VKH) syndrome, which is one of the most common diseases in the class of non-infectious uveitis, multiple serous retinal detachments and inflammation are seen as a result of autoimmunity against melanocytes [15]. In this case, the presence of multiple serous detachments, as we have seen in VKH syndrome, makes us think of a kind of Harada-like syndrome. Its different aspect from VKH is that it is a non-infectious uveitis that develops due to the toxicity of drugs, not an underlying autoimmunity. In the follow-up of the patients, it is necessary to evaluate whether the inflammation is active by measuring the choroidal thickness in each follow-up, just as in the VKH syndrome. It is also known that fundus fluorescein angiography every 3 months is useful in follow-ups. These patients should be followed up just like a VKH patient. As we know in VKH disease, primarily systemic corticosteroids are used in the treatment; since it is an autoimmune disease, various antimetabolites and biological agents can also be used [16]. However, as in our patient -uveitis caused by drug use-, this type of immune-mediated drugs cannot be used because there is an underlying oncological disease. In these patients, if possible, the chemotherapeutic drugs should be discontinued and systemic corticosteroid therapy should be administered.

## Conclusion

During the use of MAPK pathway inhibitors, patients must come to ophthalmology examinations regularly. Patients should also be checked out for side effects such as uveitis, serous retinal detachment, macular edema, and central retinal vein occlusion. Various systemic diseases and rheumatological diseases can cause uveitis, but it should be kept in mind that uveitis may develop as a result of ocular toxicity of some drugs.

## Data Availability

All data generated or analyzed during this study are included in this published article.
